# Hochuekkito can Prevent the Colonization of Methicillin-Resistant *Staphylococcus aureus* in Upper Respiratory Tract of Acute Stroke Patients

**DOI:** 10.3389/fphar.2021.683171

**Published:** 2021-06-28

**Authors:** Masakazu Kitahara, Shin Takayama, Tetsuya Akaishi, Akiko Kikuchi, Tadashi Ishii

**Affiliations:** ^1^ Department of Neurosurgery, Kenwakai Hospital, Iida, Japan; ^2^ Department of Education and Support for Regional Medicine, Tohoku University Hospital, Sendai, Japan; ^3^ Department of Kampo Medicine, Tohoku University Hospital, Sendai, Japan; ^4^ Department of Kampo and Integrative Medicine, Tohoku University Graduate School of Medicine, Sendai, Japan

**Keywords:** methicillin-resistant *Staphylococcus aureus*, prevention, colonization, Hochuekkito, Kampo medicine

## Abstract

**Background:** Methicillin-resistant *Staphylococcus aureus* (MRSA) colonization can lead to MRSA pneumonia or other infections in compromised hosts, and invasive MRSA infections lead to significant morbidity and mortality. The present observational study elucidated whether administration of hochuekkito (HET) can prevent MRSA colonization in the upper respiratory tract and support recovery in acute stroke patients.

**Methods:** In this retrospective, observational study, 73 acute stroke patients admitted to Kenwakai Hospital between April 2007 and December 2019 who did not require emergency surgery during this period were enrolled. Conventional treatment was provided to all patients, depending on their condition, and 7.5 g/day of HET was administered to the patients who could take the medicine via nasogastric tube or orally in three divided doses for three months. Bacterial cultures from laryngeal swabs and sputum were evaluated every week. We evaluated the presence of MRSA infection or another infectious disease within 30 days of admission; modified Rankin Scale scores, which assesses the independent living skills after stroke at three months after admission; and blood biomarkers (white blood cell count, albumin levels, C-reactive protein levels, and hemoglobin levels).

**Results:** In total, 73 patients (HET group, *n* = 41; non-HET group, *n* = 32) were enrolled in the study. MRSA detection was significantly less likely in the HET group than in the non-HET group (*p* = 0.0497). The incidence of infectious diseases was significantly lower in the HET group than in the non-HET group (*p* = 0.0096), and the modified Rankin Scale score at three months was also significantly lower in the HET group than in the non-HET group (*p* = 0.033). The white blood cell count, and serum C-reactive protein levels did not differ between those who were treated with HET and those who were not. However, serum albumin and hemoglobin levels improved slightly between month one and month three after admission only in those who were treated with HET.

**Conclusion:** Our results indicate that the administration of HET may contribute to the prevention of MRSA colonization and promote rehabilitation in stroke patients.

## Introduction

Methicillin-resistant *Staphylococcus aureus* (MRSA) is a common cause of healthcare-associated infections and is the most frequently isolated resistant bacteria in hospitals. Although the frequency of isolation differs among the hospitals, it has been estimated that MRSA accounts for 50–70% of all confirmed cases of *S. aureus* infection among the hospitalized patients in Japan (Ministry of Health, Labour and Welfare, 2018). The incidence of MRSA infection has been estimated to be 10 per 100,000 people (Tanihara and Suzuki, 2016). MRSA infections are categorized as hospital-associated, community-acquired, and livestock-associated MRSA. Many people are the carriers of MRSA; and in these individuals, care is required to avoid transmission to the compromised hosts. MRSA is particularly problematic because it can cause intractable infections in hospitalized patients and can also cause outbreaks in the hospitals. Invasive MRSA infections lead to significant morbidity and mortality (Inagaki et al., 2019). MRSA bacteremia is associated with significant healthcare costs, morbidity, and mortality (Ortwine and Bhavan, 2018). Prevention of the spread of MRSA is, therefore, important to control the MRSA infection rates (Hansen et al., 2010). MRSA can easily colonize the nasal and upper respiratory tract in the compromised hosts, especially in patients with acute brain injuries, such as cerebral infarction and cerebral bleeding. Brain damage can inhibit the swallowing reflex and cough reflex, which can lead to aspiration bronchitis and pneumonia. Most of the problems associated with the prevention of MRSA infection relate to the prevention of MRSA colonization.

Six anti-MRSA drugs, viz., vancomycin, teicoplanin, arbekacin, linezolid, daptomycin, and tedizolid, have been approved in Japan. Some bacteria can be resistant to all of the aforementioned drugs; therefore, they should be used with caution. It is not recommended to use the anti-MRSA drugs for MRSA carriers.

In Japan, Kampo medicine has been used to promote innate immunity. In particular, Hochuekkito (HET) has been used to promote immunity, nutrition, physical strength, and recovery from the compromised conditions. HET is widely used for the relief of impaired constitution during the recovery from diseases, fatigue, malaise, and anorexia in the national health insurance system; it has also been introduced into some clinical practice guidelines in Japan (Takayama and Iwasaki, 2017; Takayama et al., 2018; Takayama et al., 2020a). HET is a multifunctional drug composed of 10 crude drugs, and it has been documented to promote appetite, support nutrition, and have anti-infection and anti-inflammatory properties (Takayama et al., 2020b). These effects can help prevent colonization in a compromised host.

The present observational study was conducted in patients with and without the administration of HET to investigate the contribution of HET to the prevention of MRSA colonization.

## Materials and Methods

### Patients

The patients admitted to Kenwakai Hospital between April 2007 and December 2019 with impaired consciousness secondary to acute stroke, including cerebral infarction and hemorrhage, were enrolled in the study. The hospital is located in the Nagano prefecture of Japan and provides acute stroke care from the emergency admission to rehabilitation. The data of 73 consecutive patients who were hospitalized with acute stroke and who did not require emergency surgery during this period were collected. The patients with gastrointestinal bleeding, severe diarrhea, and in whom there was a difficulty in insertion of the nasogastric tube were not included in the study. Conventional treatment was provided to all patients, depending on their condition, and 7.5 g/day of HET was administered to the patients who could take the medicine via nasogastric tube or orally in three divided doses for three months. No patient received antibiotics before the detection of infectious diseases.

This observational study was approved by the institutional review board of the Tohoku University Graduate School of Medicine (Institutional Review Board No. 20122). Informed consent was not obtained from the research subjects. However, information about the research, including its purpose, were made available to the public, and subjects were allowed to refuse participation. Disclosure of research-related matters was made by posting disclosure materials on the website or hospital bulletin board of each institution. The principal investigator or researcher responded to any inquiries.

### Hochuekkito

HET extract produced by NIPPON FUNMATSU YAKUHIN Co., LTD (Tokyo, Japan) contained 10 crude drugs: The Japanese Pharmacopoeia (JP) Astragalus root [*Astragalus membranaceus* Bunge, or *Astragalus mongholicus* Bunge (*Leguminosae*), *radix*], JP Atractylodes rhizome [*Atractylodes japonica* Koidzumi ex Kitamura, or *Atractylodes macrocephala* Koidzumi (*Atractylodes ovata* De Candolle) (*Asteraceae*), *rhizoma*], JP Ginseng [*Panax ginseng* C. A. Meyer (*Panax schinseng* Nees) (*Araliaceae*), *radix*], JP Japanese angelica root [*Angelica acutiloba* Kitagawa, or *Angelica acutiloba* Kitagawa var. *sugiyamae* Hikino (*Apiaceae*), *radix*], JP Bupleurum root [*Bupleurum falcatum* Linné (*Apiaceae*), *radix*], JP Jujube [*Zizyphus jujuba* Miller var. *inermis* Rehder (*Rhamnaceae*), *fructus*], JP Citrus unshiu peel [*Citrus unshiu* Marcowicz, or *Citrus reticulata* Blanco (*Rutaceae*), *pericarpium*], JP Glycyrrhiza [*Glycyrrhiza uralensis* Fischer, or *Glycyrrhiza glabra* Linné (*Leguminosae*), *radix*], JP Cimicifuga rhizome [*Cimicifuga simplex* Turczaninow, *Cimicifuga dahurica* Maximowicz, *Cimicifuga foetida* Linné, or *Cimicifuga heracleifolia* Komarov (*Ranunculaceae*), rhizoma], and JP Processed ginger [*Zingiber officinale* Roscoe (*Zingiberaceae*), *rhizoma processum*]. HET include the index components of hesperidin from 23 to 27 mg/5g, saikosaponin B2 from 0.7 to 1.0 mg/5g, and glycyrrhizic acid from 20 to 25 mg/5g.

### Studied Variables

The following demographic and clinical data of 73 patients with acute stroke were collected: sex; age; primary disease; history of diabetes mellitus; history of hypertension; other medical history; worst Japan Coma Scale score; confirmed detection of MRSA in culture during the first 30 days of hospital admission; confirmed infectious disease during the first 30 days of hospital admission; modified Rankin Scale (mRS) score, which assesses the independent living skills after stroke and before admission; mRS score on admission; mRS score at three months after admission; and oral food intake ability at three months after admission. Bacterial cultures from laryngeal swabs and sputum were evaluated every week. In terms of blood biomarkers, white blood cell (WBC) count, albumin levels, C-reactive protein levels, and hemoglobin levels at the time of initial admission, at one month, and three months from hospitalization were evaluated.

### Statistical Analyses

Comparisons of the distribution of a variable between two groups were performed with the Student’s *t*-test or Mann–Whitney *U* test according to the distribution pattern of the studied variable. Changes in the variables between the three time points were evaluated by repeated measures analysis of variance followed by Bonferroni post hoc test. Comparisons of the frequency of a variable between two groups were performed using the chi-square test or Fisher’s exact test according to the sample size of each cell. After the univariate analyses, multivariate analyses with binary logistic regression analyses were performed to identify the predictors of MRSA colonization. In the multivariate analyses, variables of particular clinical interest and other variables that showed significant predictive impact (*p* < 0.10) on MRSA detection in the univariate analyses were included as the explanatory variables. In each analysis, *p* values < 0.05 were considered statistically significant. Statistical analyses were performed using SPSS Statistics software, version 22 (IBM Corp., Armonk, NY, United States) or MATLAB R2015a (MathWorks, Natick, MA, United States).

## Results

### Clinical Data

**FIGURE 1:**
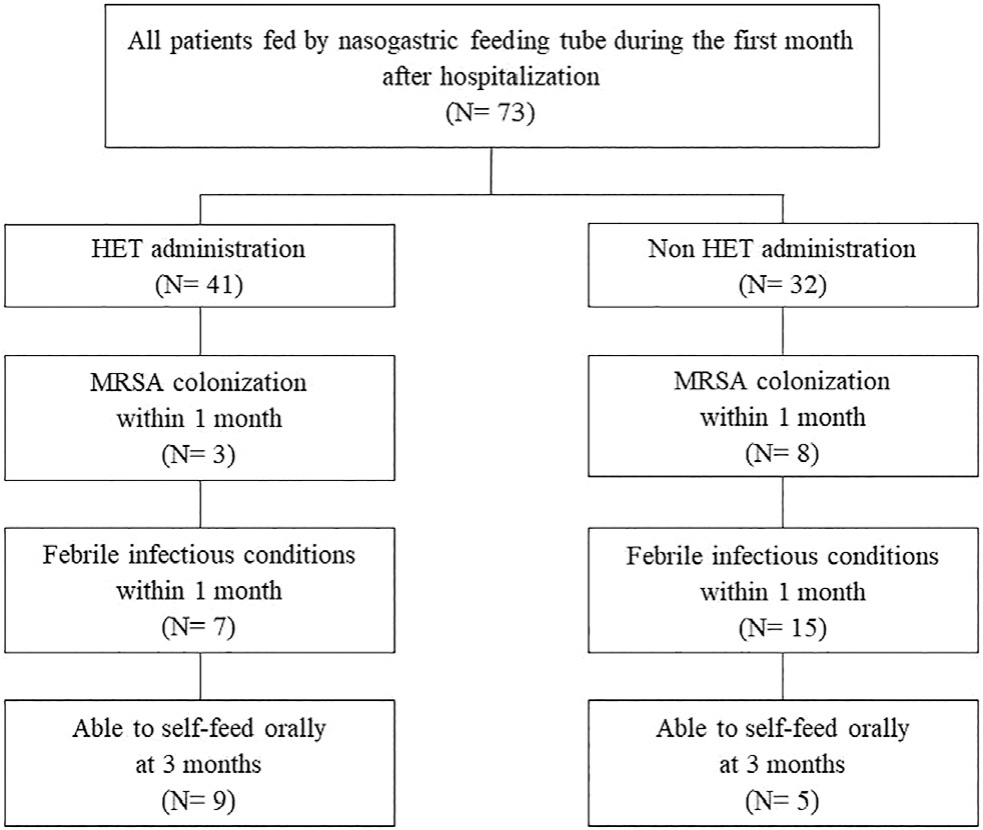
Flowchart depicting the study participants.

A flowchart depicting the study participants is shown in Figure 1. In total, 73 patients (HET administration group, *n* = 41; HET non-administration group, *n* = 32) were enrolled. All patients were fed by nasogastric feeding tube during the first month after hospitalization. Of these patients, 14 (HET administration group, *n* = 9; HET non-administration group, *n* = 5) were able to self-feed orally after 2 months. Demographic and clinical characteristics of the patients are listed in Table 1. The rate of MRSA detection, the incidence of infectious diseases, and the mRS score at 3 months were significantly lower in the HET group than in the non-HET group (*p* = 0.0497, 0.0096, and 0.033, respectively).

**TABLE 1:** Demographic and clinical characteristics of the patients in HET and non-HET groups.

	HET group (*n* = 41)	Non-HET group (*n* = 32)	*p*-value
Male:Female (*n*)	12:29	18:14	0.0201
Age (years)	83.0 ± 9.2	82.2 ± 10.2	0.72
Diabetes mellitus, *n* (%)	4 (9.8%)	7 (21.9%)	0.19
Hypertension, *n* (%)	30 (73.2%)	18 (56.3%)	0.13
MRSA colonization within 1 month, *n* (%)	3 (7.3%)	8 (25.0%)	0.0497
Febrile infectious conditions within 1 month, *n* (%)	7 (17.1%)	15 (46.9%)	0.0096
Worst JCS score * (0: best; 300: worst)	30 (20–200)	20 (3–30)	0.0029
mRS score before admission	0.81 ± 0.90	0.81 ± 0.86	0.92
mRS score on admission	5.00 ± 0.00	5.00 ± 0.00	1.00
mRS score at 3 months (0: best; 6: worst)	4.66 ± 0.88	5.06 ± 0.44	0.0329
Enteral tube feeding:Oral intake (*n*)			
On admission	41:0	32:0	1.00
At 1 month	41:0	32:0	1.00
At 3 months	32:9	27:5	0.56

Except where noted, continuous variables are presented as mean ± standard deviation.

* Median (interquartile range).

Abbreviations: HET, hochuekkito; JCS, Japan Coma Scale; mRS, modified Rankin Scale; MRSA, methicillin-resistant *Staphylococcus aureus*.

### Risk Factors of MRSA Colonization in the First 1 month

To identify the risk factors for MRSA colonization within the first month of hospitalization, please refer to Table 2, which displays the clinical data of the enrolled 73 patients, stratified by the MRSA colonization status. The rate of HET administration was lower among those who were later found to have MRSA colonization (27.3 vs. 61.3%; *p* = 0.0497).

**TABLE 2:** Patients’ characteristics stratified by MRSA colonization status in the first month.

	MRSA (+) within 1 month (*n* = 11)	MRSA (−) (*n* = 62)	*p*-Values
Male:Female (*n*)	5:6	25:37	0.75
Age (years)	81.9 ± 13.2	82.8 ± 8.9	0.78
Diabetes mellitus, *n* (%)	4 (36.4%)	7 (11.3%)	0.0545
Hypertension, *n* (%)	9 (81.8%)	39 (62.9%)	0.31
HET administration, *n* (%)	3 (27.3%)	38 (61.3%)	0.0497
Febrile infectious conditions within 1 month, *n* (%)	3 (27.3%)	18 (29.0%)	1.00
Worst JCS score * (0: best; 300: worst)	30 (20–100)	20 (3–100)	0.39

Except where noted, continuous variables are presented as mean ± standard deviation.

* Median (interquartile range).

Abbreviations: HET, hochuekkito; JCS, Japan Coma Scale; MRSA, methicillin-resistant *Staphylococcus aureus*.

### Multivariate Analysis to Identify the Risk Factors for MRSA Colonization

Based on the aforementioned univariate analyses, binary logistic regression analyses evaluating the risk factors for the later detection of MRSA colonization were performed using the data of all patients (*n* = 73). The results of the multivariate analysis are presented in Table 3. Only the history of HET administration was a significant predictor of later MRSA colonization.

**TABLE 3:** Multivariate analyses of the predictors for MRSA colonization.

	Odds ratio (95% CI)	*p*-Values
Sex	0.598 (0.126–2.839)	0.52
Age	0.984 (0.910–1.064)	0.68
HET administration	0.147 (0.026–0.819)	0.0287
Hypertension	4.793 (0.708–32.440)	0.11
Diabetes mellitus	3.234 (0.617–16.957)	0.17
JCS at worst	1.004 (0.992–1.016)	0.51

The odds ratios and *p*-values are the results of binary logistic regression analyses using the detection of MRSA colonization as the objective variable.

Abbreviations: CI, confidence interval; HET, Hochuekkito; JCS, Japan Coma Scale; MRSA, methicillin-resistant *Staphylococcus aureus*.

### Changes in Blood Biomarkers Following the Administration of HET

**FIGURE 2:**
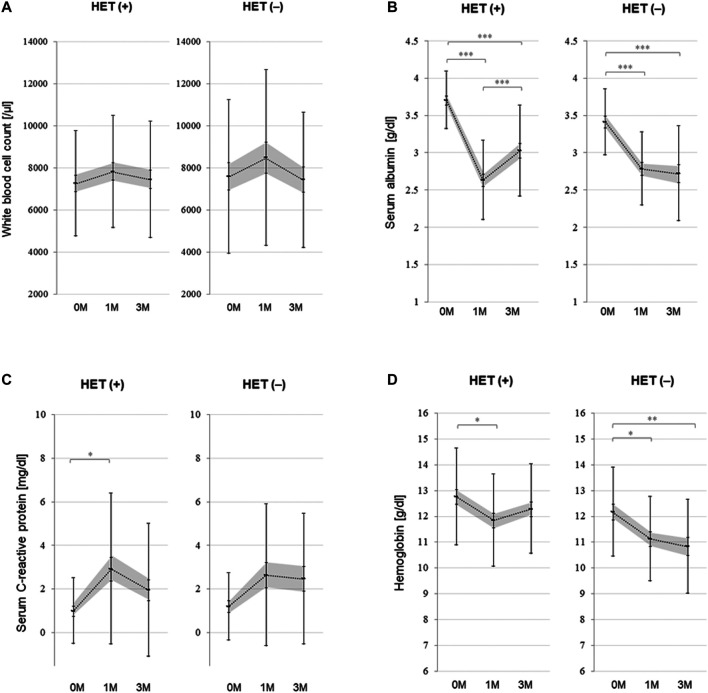
Changes in the blood biomarker levels in the patients of HET and non-HET groups. Changes in the white blood cell count **(A)**, serum albumin level **(B)**, serum C-reactive protein level **(C)**, and hemoglobin level **(D)** in the patients of HET and non-HET groups. The dotted lines show the change in average. Error bars show the standard deviations, and gray colored areas show the range of average ± standard error. Abbreviations: HET, hochuekkito; M, month(s) *p < 0.01, **p < 0.001, ***p < 0.0001.

In order to identify the underlying reasons for a decreased MRSA colonization rate and better mRS scores at three months after hospitalization in those treated with HET, we evaluated the changes in the blood biomarkers. As shown in Figure 2, the WBC count and serum C-reactive protein levels did not differ between those who were treated with HET and those who were not. However, serum albumin and hemoglobin levels improved slightly between month one and month three after admission only in those who were treated with HET.

## Discussion

In the present study, administration of HET was associated with a significantly reduced occurrence of MRSA colonization and development of new infectious diseases. The history of HET administration significantly predicted subsequent MRSA colonization. Furthermore, HET administration was also associated with a significantly improved functional independence score.

A few studies have shown the effect of Kampo medicine on decolonization of MRSA in patients. Nishida (2003) reported that HET administration significantly reduced the bacterial count in urine culture at 10 weeks after administration in asymptomatic patients. Karibe et al. (1997) reported that the administration of Kampo medicines, HET and juzentaihoto, significantly reduced the rate of MRSA among MRSA carriers in the neurosurgery unit. Nakae et al. (2013) also reported that juzentaihoto administration led to negative bacterial cultures in five patients with refractory multidrug-resistant bacterial infections, including MRSA, with severe burns. In an experimental study, Matsushima et al. (2012) reported that HET administration inhibited the growth of MRSA and extended survival times in MRSA-infected mice. However, to the best of our knowledge, no previous study has revealed the preventive effect of Kampo medicine on MRSA colonization; therefore, this is the first report to elucidate the preventive effect of HET administration on MRSA colonization.

The present study showed that HET administration was associated with a significant improvement in the functional independence score in acute stroke patients at three months after admission. Furthermore, the HET group showed slightly improved serum albumin and hemoglobin levels between month one and month three after admission, which was not observed in the non-HET group. However, inflammatory marker levels did not differ between the groups. These results imply that the effect of HET may result from improved levels of serum nutritional marker rather than the changes in inflammatory marker levels.

With regard to appetite, nutrition, and chronic inflammation, Shinozuka et al. (2007) and Tatsumi et al. (2009) reported that HET administration decreased the serum C-reactive protein, tumor necrosis factor-α, and interleukin-6 levels and increased the serum prealbumin levels, with a significant increase in body weight and improvement in the quality of life in patients with chronic obstructive pulmonary disease. Fukumura et al. (2017) reported that HET administration reduced the incidence of inflammatory complications in patients with concomitant cerebrovascular diseases in convalescent rehabilitation. The report also suggested that HET administration reduced the development of infectious diseases and promoted rehabilitation.

HET includes 10 crude drugs: JP *Astragalus* root, JP *Atractylodes lancea* rhizome, JP ginseng, JP Japanese angelica root, JP *Bupleurum* root, JP jujube, JP *Citrus unshiu* peel, JP *Glycyrrhiza*, JP *Cimicifuga* rhizome, and JP ginger. The pharmacological mechanism of action is likely to be complex because HET includes multiple pharmacologically active substances; however, its immuno-stimulatory effects against infection, immuno-modulatory effects against inflammation, and ameliorative effects against exhaustion and frailty have been reported in pharmacological studies and clinical studies (Takayama et al., 2020b). To prevent MRSA colonization, a good level of consciousness, ability to self-administer the oral intake, appropriate nutrition as well as innate immunity, and contact transmission are important. Consistent with the previous reports, our findings suggest that HET can support self-administered oral intake, nutrition, and innate immunity, and suppress inflammation. These mechanisms may underlie the prevention of MRSA colonization.

Our study has a few limitations. First, this is a retrospective observational study; thus, the characteristics of patients could not be controlled for. The data of the patients with and without HET were compared in the present study. The results showed a significant improvement in mRS and the ability of oral intake at three months after admission between the groups. Second, we did not prescribe HET for patients with gastroduodenal bleeding, diarrhea, and those who experienced difficulty in nasogastric tube insertion; thus, the condition of tube feeding might have influenced the results. To confirm the actual effect of HET on the prevention of MRSA colonization, further randomized controlled trials are required. Finally, this study was conducted as a part of the routine clinical practice, and the blood sampling for the estimation of cytokine levels was not performed.

MRSA colonization can lead to MRSA pneumonia or other infections in compromised hosts. Nosocomial infection with MRSA is a risk factor for outbreak accidents in hospitals. The findings of the present study suggest that HET administration may contribute to the prevention of MRSA colonization and promote rehabilitation in stroke patients.

## Data Availability

The raw data supporting the conclusions of this article will be made available by the authors, without undue reservation.

## Abbreviations

HET, hochuekkito; JCS, Japan Coma Scale; mRS, modified Rankin Scale; MRSA, methicillin-resistant *Staphylococcus aureus*; WBC, white blood cell.
